# Transposable Element-Driven Genomic Plasticity: Unveiling the Evolutionary Mechanisms of Lifestyle Transition and Ecological Adaptation in Endophytic Fungi

**DOI:** 10.3390/jof12040273

**Published:** 2026-04-09

**Authors:** Yunfeng Lai, Cunzhong Fan, Zhibin Zhang, Riming Yan, Du Zhu, Huilin Yang

**Affiliations:** 1 Key Laboratory of Biodiversity Conservation and Bioresource Utilization of Jiangxi Province, College of Life Science, Jiangxi Normal University, Nanchang 330022, China; 2 Key Laboratory of Natural Microbial Medicine Research of Jiangxi Province, College of Life Science, Jiangxi Science and Technology Normal University, Nanchang 330013, China

**Keywords:** endophytic fungi, comparative genomics, evolutionary mechanisms, secreted proteins, virulence factors

## Abstract

The genomic basis underlying the remarkable ecological flexibility of endophytic fungi (EF), particularly their potential to transition between symbiotic, saprophytic, and pathogenic lifestyles, remains poorly understood. Through comparative genomics of 75 Ascomycota and a validation set of 36 *Fusarium* genomes, we uncovered a distinct pattern of genome evolution in EF, contrasting with the well-known “gene loss” model in obligate symbionts. Our analysis reveals that EF genomes are significantly expanded, primarily driven by the accumulation of DNA transposable elements (TEs). Crucially, this TE-mediated genomic plasticity is coupled with the retention and significant expansion of gene families for both saprotrophy and potential pathogenesis. We propose a novel “dual-trophic potential” model: TE-driven genomic expansion and plasticity provide the genetic raw material for EF to maintain a versatile repertoire of ecological tools, facilitating adaptive shifts across the endophytic–saprophytic–pathogenic continuum. This study reframes our understanding of fungal endophytism from a static symbiotic state to a dynamic, genetically enabled ecological strategy.

## 1. Introduction

Fungi play crucial ecological roles in maintaining ecosystem stability through complex interaction networks with plants, enabling multidimensional regulation. Based on lifestyle, fungi are classified into endophytic fungi (EF), phytopathogenic fungi (PF), and saprophytic fungi (SF). EF colonize internal plant tissues during at least a portion of their life cycle, typically without inducing visible disease symptoms [1]. This endophytic lifestyle is characterized by high phenotypic plasticity, enabling these fungi to adapt dynamically to fluctuating host conditions and environmental cues, with some strains even exhibiting the capacity to transition to pathogenic or saprophytic states [2]. They form mutualistic symbiotic relationships with their host plants and serve crucial regulatory functions in enhancing abiotic stress tolerance and synthesizing secondary metabolites [3,4,5]. PF mediate plant pathogenesis through virulence factors [6,7]. SF degrade soil organic matter by secreting diverse hydrolytic enzymes such as lignin peroxidases and cellulases [8,9]. However, these lifestyle classifications are not absolute. Fungi identified as endophytes under one set of conditions may exhibit pathogenic or saprophytic behavior in other contexts, reflecting a fundamental phenotypic plasticity that characterizes many fungal species [10,11].

Transposable elements (TEs) are mobile genetic elements capable of changing their positions within the genome, thereby exerting profound effects on gene function, genome structure, and biological evolution. TEs constitute a significant portion of fungal genomes. Their insertion into the genome not only expands genome size but also disrupts gene continuity, alters gene transcription and expression, leads to chromosomal rearrangements through genetic recombination, and promotes the generation of insertional mutations. The insertion sites of TEs within host genomes are not random but exhibit notable preferences. Some TEs tend to insert into specific locations, such as promoter regions or the junctions between introns and exons of host genes [12]. These TE insertions have a dual effect on the host genome: on one hand, they may confer benefits for gene regulation and evolution; on the other hand, they pose potential risks such as gene disruption and expression interference. To protect the genome from interference by foreign TEs, hosts initiate a series of complex defense mechanisms, including DNA methylation and RNA interference [13,14]. Based on their transposition mechanisms, TEs can be classified into several types: DNA transposons, long terminal repeat (LTR) retrotransposons, and non-long terminal repeat (non-LTR) retrotransposons [15].

Although the ecological functions of endophytic fungi are well-recognized, the genomic mechanisms underlying their adaptation to an endophytic lifestyle remain unclear. Studies on ectomycorrhizal fungi, as model systems, have shown that during the evolution from saprotrophic ancestors to a symbiotic lifestyle, extensive loss of plant cell wall-degrading enzyme (PCWDE) gene families occurs. This gene loss is regarded as an adaptive trait that minimizes harm to the host and helps sustain a stable symbiotic relationship [16,17,18,19]. However, whether this “gene loss” model applies to phylogenetically broader groups of endophytic fungi remains controversial.

To elucidate the genomic adaptations of EF, we analyzed 75 Ascomycota genomes (34 EF, 29 PF, 12 SF). We performed standardized annotation and systematically compared key gene families—including carbohydrate-active enzymes (CAZymes), PCWDEs, microbial cell wall-degrading enzymes (MCWDEs), and virulence factors—in both the proteome and secretome. This approach aimed to investigate the mechanisms of endophytic adaptation and to analyze the influence of TE content, genetic background, and lifestyle on genomic characteristics. To validate the reliability and generalizability of our findings, we selected an independent dataset of 36 *Fusarium* strains (18 EF, 9 PF, 9 SF) characterized by rich genomic data and diverse lifestyles, and applied the same analytical pipeline.

## 2. Materials and Methods

### 2.1. Data Acquisition and Quality Assessment

#### 2.1.1. Data Sources

Genomic data were obtained from the National Center for Biotechnology Information (NCBI) public database. The dataset comprised two subsets: 75 Ascomycota and 36 *Fusarium* genomes (Table A1 and Table A2). Our classification of strains into EF, PF, and SF was based on their primary isolation source as recorded in NCBI BioSample records. The Ascomycota subset included 29 endophytic (EF) and 46 non-endophytic strains. The *Fusarium* subset contained 18 EF and 18 non-EF strains. Genomes were selected based on assembly completeness, assessed by Benchmarking Universal Single-Copy Orthologs (BUSCO): ≥85% for Ascomycota and ≥90% for *Fusarium* strains.

#### 2.1.2. Genome Assembly Completeness Assessment

Genome assembly completeness was assessed with BUSCO v5.8.3 [20] using the fungi_odb10 dataset. This tool assesses completeness by searching for a set of 758 conserved fungal single-copy orthologs [21]. Results are categorized as “complete” (single or duplicated), “fragmented”, or “missing” based on alignment scores. Assembly completeness is reported as the percentage of complete genes found, providing a measure of phylogenetic breadth and quality for downstream analysis [22].

### 2.2. Phylogenetic and Evolutionary Analysis

#### 2.2.1. Phylogenetic Tree Construction

Single-copy orthologs were identified with OrthoFinder v3.0.1b1 [23]. Their protein sequences were aligned with MUSCLE v5.3 [24], and poorly aligned positions were removed with Gblocks v0.91b [25] (parameters: -b5=h -t=p). A maximum-likelihood phylogenetic tree was constructed with RAxML v8.2.13 [26], and branch support was evaluated with 1000 bootstrap replicates.

#### 2.2.2. Divergence Time Estimation

Divergence times were estimated using the penalized likelihood method in r8s v1.8.1 [27], employing the POWELL optimization algorithm. Model fitting was validated by cross-validation. The Ascomycota tree was calibrated with three fossil constraints from TimeTree [28]: *C. parasitica*–*D. alcacerensis*, *P. eosporulosa*–*D. alcacerensis*, and *C. leucostoma*–*A. luchuensis*. The *Fusarium* tree was calibrated using *Akanthomyces lecanii* as the outgroup, with a fossil constraint on the *F. coicis*–*A. lecanii* divergence.

### 2.3. Genome Annotation

#### 2.3.1. Repetitive Sequence Annotation

De novo repeat libraries were constructed for the 75 Ascomycota and 36 *Fusarium* genomes separately using RepeatModeler v2.0.6 [29]. The analysis integrated the RECON [30] and RepeatScout modules for comprehensive repeat detection. Tandem repeats were identified with Tandem Repeats Finder (TRF) v4.09.1 [31], and LTR retrotransposons were detected using the built-in LTR discovery function. Finally, all genomes were masked and annotated for repetitive elements using RepeatMasker v4.0.9 [32] against the custom libraries, yielding their genomic coverage and composition.

#### 2.3.2. Gene Prediction and Functional Annotation

Protein-coding genes were predicted de novo using GeneMark-ES v4.33 [33], which employs a self-training hidden Markov model (HMM) for consistency. Functional annotation was performed as follows: Proteases were annotated using MEROPS [34]. Virulence factors were assigned based on the PHI [35] and DFVF [36] databases. CAZyme families were annotated using dbCAN v4.1.4 [37]. Secreted proteins were predicted with a sequential pipeline: signal peptides were predicted with SignalP v5.0 [38]; proteins with transmembrane domains were removed using TMHMM v2.0c [39]; GPI-anchored proteins were filtered out with PredGPI [40]; and extracellular localization was confirmed with BUSCA [41].

### 2.4. Statistical Analysis

Differences in genomic features and functional gene content across lifestyles were tested using Permutational Multivariate Analysis of Variance (PERMANOVA) implemented in the RVAideMemoire R package, with Jaccard distances [42]. All PERMANOVA analyses were performed using the adonis2 function in the R package vegan with 999 permutations. Model assumptions, including homogeneity of multivariate dispersion, were assessed using the betadisper function; no significant violations were detected (*p* > 0.05 for all comparisons). For each factor, we report the proportion of variance explained (R^2^, effect size) and the associated *p*-value. Where applicable, 95% confidence intervals for effect sizes were estimated via bootstrap resampling (1000 iterations). Pairwise comparisons among lifestyle groups were corrected for multiple testing using the false discovery rate method.

The effects of phylogeny, lifestyle, and TE content on genomic features were assessed using PERMANOVA (vegan package in R), with phylogenetic distances calculated from the alignment (Biopython Phylo module). Significance and effect sizes were based on *p*-values and R^2^ [43]. The first two principal coordinates derived from the phylogenetic distance matrix (referred to as PhyloDist.PC1 and PhyloDist.PC2) represent the phylogenetic structure. Figures were generated using R packages including ggplot2, ggpubr, and pheatmap.

## 3. Results

### 3.1. Phylogenetic Analysis and Divergence Time Estimation

**Figure 1:**
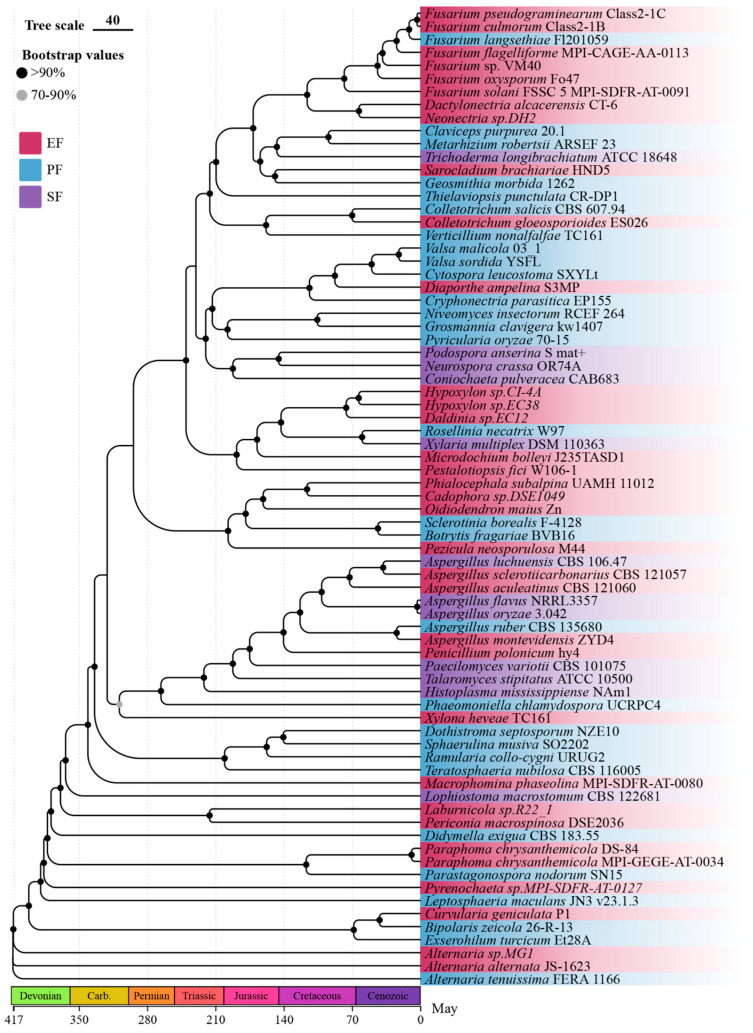
Phylogeny and divergence times of the 75 Ascomycota strains. Divergence times were estimated using r8s and are shown at the nodes. The geological timescale is indicated on the x-axis.

We reconstructed a robust maximum-likelihood phylogeny of the 75 Ascomycota strains based on 448 single-copy orthologs, with strong bootstrap support for most nodes (Figure 1). The tree reveals that endophytic fungi are phylogenetically dispersed across multiple orders (e.g., Hypocreales, Helotiales, Pleosporales), rather than forming a monophyletic group. Notably, certain clades exhibit a predominance of a single lifestyle: for instance, a lineage within the Hypocreales is composed almost entirely of pathogens, while a clade in the Helotiales consists exclusively of EFR. This scattered distribution suggests multiple independent evolutionary origins of endophytism and underscores the importance of phylogenetic context in interpreting lifestyle-associated genomic features. Divergence time estimates indicate that major radiations occurred during the Cretaceous and Paleogene, consistent with previous estimates.

**Figure 2:**
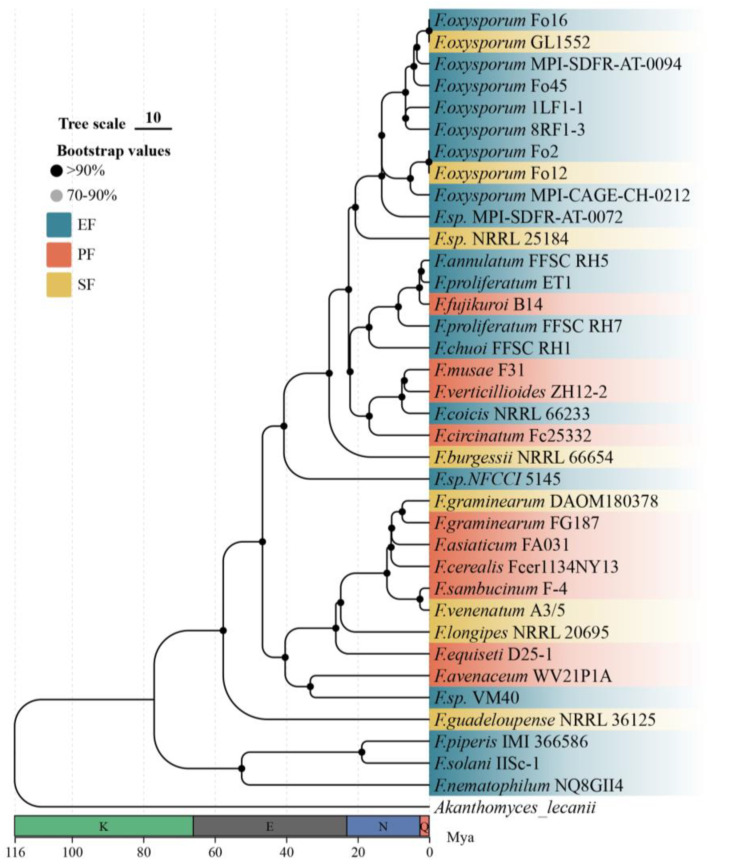
Phylogeny and divergence time of the 36 *Fusarium* strains. Divergence times were estimated using r8s. Geological periods are abbreviated on the x-axis: K, Cretaceous; E, Paleogene; N, Neogene; Q, Quaternary.

As an independent validation set, we analyzed 36 *Fusarium* genomes selected for their high assembly quality (BUSCO > 90%) and well-documented lifestyle annotations (Table A2). The use of this phylogenetically constrained genus allows us to test whether the patterns observed across Ascomycota are recapitulated within a closely related lineage, thereby mitigating the influence of deep phylogenetic divergence. From these genomes, we identified 5471 single-copy orthologous genes. Using *Akanthomyces lecanii* as an outgroup, the resulting maximum-likelihood phylogenetic tree showed exceptionally high support (all nodes >99% bootstrap; Figure 2). Divergence time estimation suggested that the genus Fusarium diversified approximately 58 million years ago (Mya) during the Paleogene period.

### 3.2. Basic Genomic Characteristics and Transposable Element Analysis

#### 3.2.1. Genome Size and Assembly Quality

We obtained genomic data for 75 Ascomycota and 36 *Fusarium* strains from the NCBI public database. The 75 Ascomycota strains spanned 17 orders and comprised 29 PF, 12 SF, and 34 EF. The EF group included 14 root endophytic fungi (EFR) and 20 non-root endophytic fungi (EFNR). The 36 *Fusarium* strains consisted of 10 PF, 8 SF, and 18 EF (with 9 EFR and 9 EFNR each).

**Figure 3:**
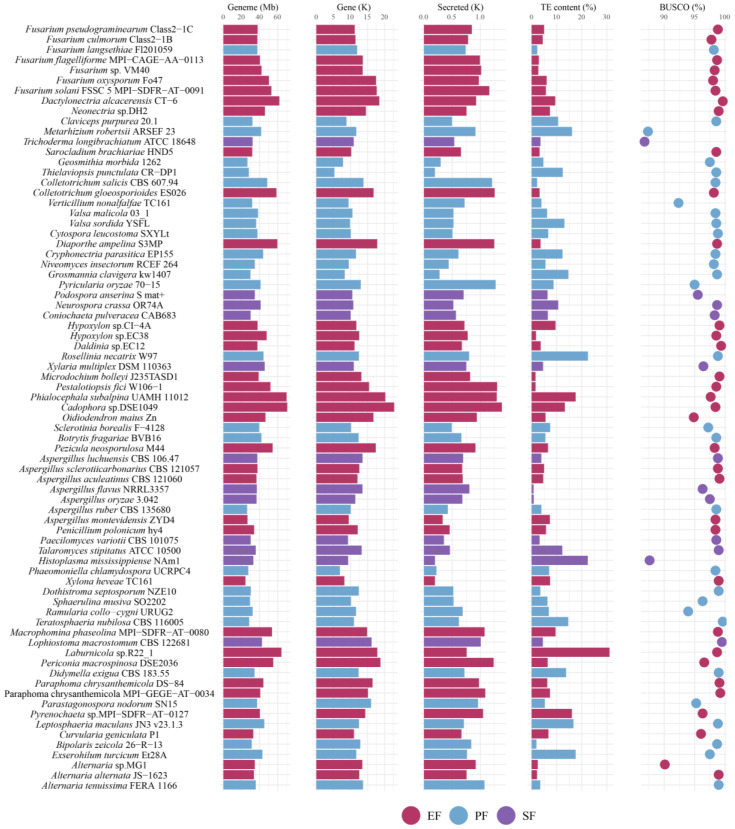
Genomic and assembly features of the 75 Ascomycota strains. Colors represent different fungal lifestyles.

**Figure 4:**
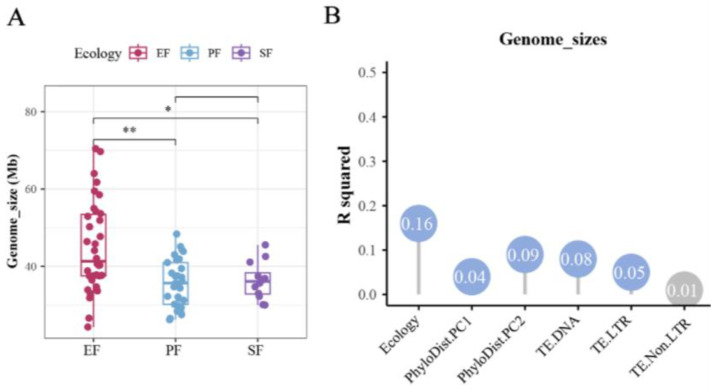
Genomic features associated with genome size variation. (**A**) Genome sizes across different fungal lifestyles (* *p* < 0.05, ** *p* < 0.01; pairwise PERMANOVA); (**B**) contribution of selected genomic features to genome size variation (R^2^). Blue circles denote variables with a significant effect (*p* < 0.05, pairwise PERMANOVA). Abbreviations: PhyloDist.PC1–2, principal components capturing >80% of phylogenetic distance variation; Ecology, the three fungal lifestyles; TE.DNA/LTR/Non.LTR, coverage of DNA transposons, LTR retrotransposons, and non-LTR retrotransposons, respectively.

Among the 75 Ascomycota strains, the number of predicted protein-coding genes ranged from 5296 to 22,762. BUSCO assessment revealed that assembly completeness exceeded 85% for all genomes, and 96% of genomes had completeness above 90% (Figure 3). Although assembly continuity varied, genome completeness showed low variation (coefficient of variation, c.v. = 2.73%). The genome size of EF was significantly larger than that of PF and SF (adjusted false discovery rate: *p* < 0.05, pairwise PERMANOVA; Figure 4A). Genome sizes varied significantly across different orders. The largest genome belonged to the Helotiales root endophyte *Cadophora* sp. DSE1049 (70.46 Mb), while others ranged from 24.34 to 69.70 Mb. Genome size was influenced by phylogeny, TE content, and lifestyle, with lifestyle being the primary factor (contribution rate: 19%, *p* < 0.05, pairwise PERMANOVA; Figure 4B).

**Figure 5:**
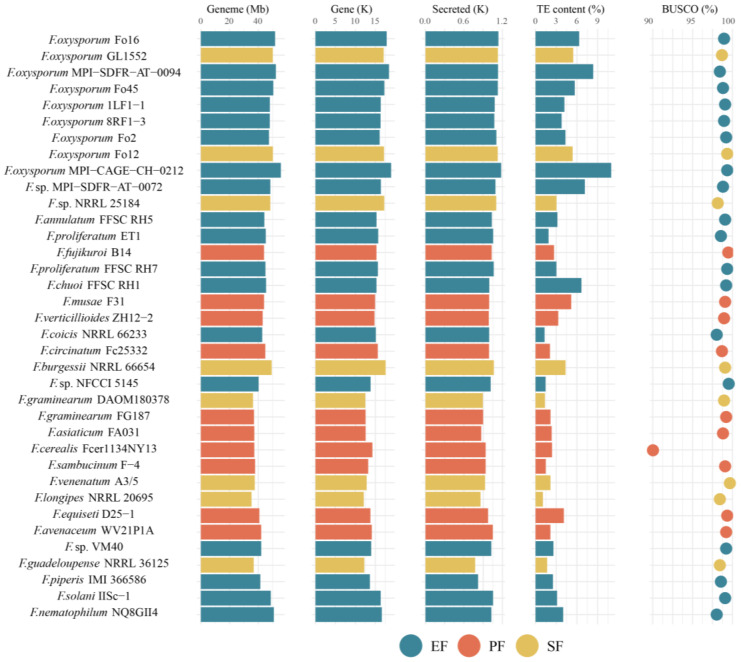
Genomic and assembly features of 36 *Fusarium* strains.

**Figure 6:**
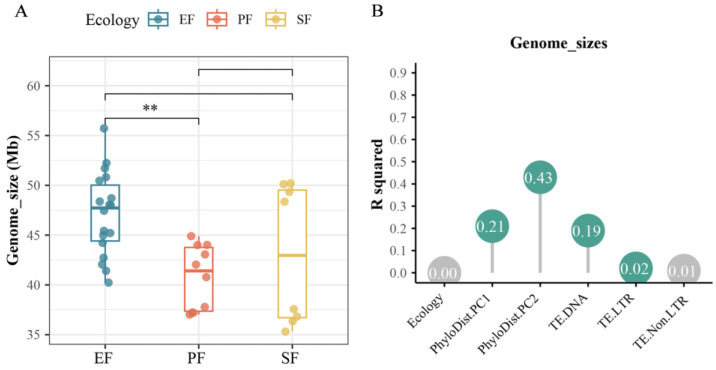
Drivers of genome size variation in *Fusarium*. (**A**) Genome size across lifestyles (** *p* < 0.01; pairwise PERMANOVA); (**B**) contribution of genomic features to genome size variation.

For the 36 *Fusarium* strains, the number of predicted coding genes ranged from 12,110 to 18,957. According to the BUSCO assessment, all genomes had an assembly completeness above 90%, with 97.2% exceeding 98% completeness (Figure 5). Although assembly continuity varied, genome completeness was highly consistent (c.v. = 1.55%). The genome size of EF was significantly larger than that of the other two fungal types (*p* < 0.05, pairwise PERMANOVA; Figure 6A), and EFR had the largest genomes among all. Phylogeny and TE content significantly influenced genome size, with phylogeny being the main contributing factor (contribution rate: 63.8%, *p* < 0.05, pairwise PERMANOVA; Figure 6B).

#### 3.2.2. Transposable-Element Content and Distribution

**Figure 7:**
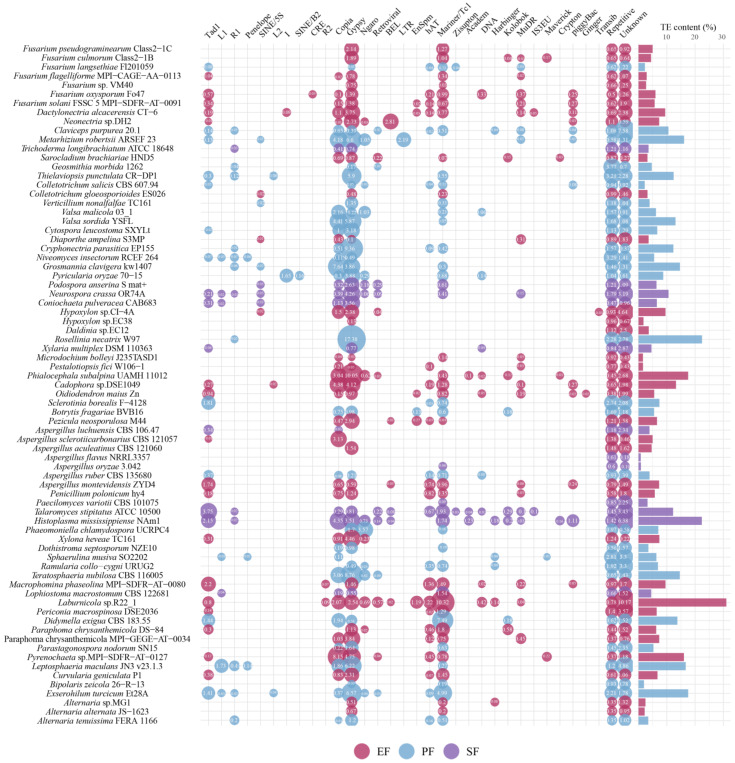
Composition and genomic coverage of transposable element families in the 75 Ascomycota strains. The size of the circle represents the magnitude of coverage.

Across the 75 Ascomycota genomes, TE content ranged from 0.79% to 31.06%. The predominant non-LTR transposon was Tad1. LTR transposons were chiefly *Copia* and *Gypsy*, and the major DNA transposon was *Mariner/Tc1* (Figure 7). Although overall TE content did not differ significantly among the three fungal lifestyles, EF exhibited a higher average content. Specifically, EFR had significantly higher DNA transposon content than PF (*p* < 0.05, pairwise PERMANOVA). This suggests that TE accumulation contributes to genome expansion in EF. Notably, *MuDR* DNA transposons were detected in all EF strains but only in a minority of PF strains, partially explaining the higher DNA transposon content in EFR. It is important to note that the observed associations between TE content and gene family expansions are correlational. While these patterns are consistent with the hypothesis that TE activity contributes to genomic plasticity and functional diversification, causal mechanisms remain to be tested through functional studies.

**Figure 8:**
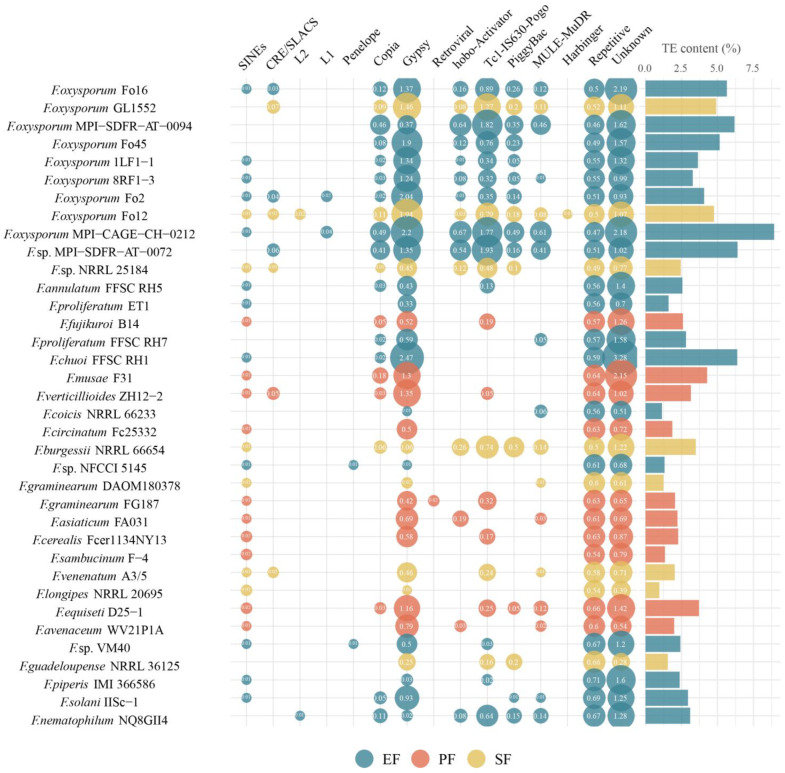
Composition and genomic coverage of transposable element families in the 36 *Fusarium* strains. The size of the circle represents the magnitude of coverage.

In the 36 *Fusarium* strains, the major transposon types were LTR retrotransposons (*Copia* and *Gypsy*) and DNA transposons (e.g., *hobo-Activator*, *Tc1-IS630-Pogo*, *PiggyBac*, and *MULE-MuDR*). TE content in these genomes ranged from 1.07% to 10.96% (Figure 8). Although TE content did not differ significantly across lifestyles, EF had a higher average content. EFR exhibited the highest average TE content, which was significantly greater than that of PF. EFR had the highest average DNA transposon content, whereas EFNR had the lowest. Specifically, DNA transposon content was significantly higher in EFR than in PF and EFNR, and also higher in SF than in PF and EFNR (*p* < 0.05, pairwise PERMANOVA). Variation in TE content was primarily influenced by LTR and DNA transposons (contribution rates: 28.0% and 20.0%, respectively), with phylogeny also contributing significantly.

### 3.3. Analysis of Functional Gene Family Expansion

#### 3.3.1. Secretome Characteristics

**Figure 9:**
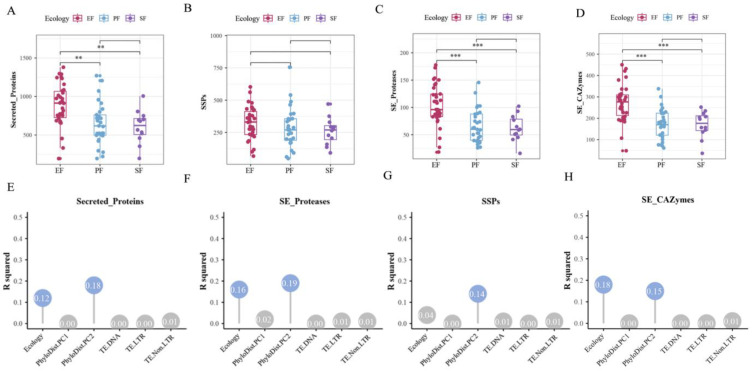
Expansion of the secretome in Ascomycota endophytes and its drivers (**, *p* < 0.01; ***, *p* < 0.001; pairwise PERMANOVA). Abundance of secreted protein (**A**), secreted protease (**B**), SSP (**C**), and secreted CAZyme (**D**) gene families across lifestyles; (**E**–**H**) contribution of genomic features to the variation in each corresponding gene family above.

Secreted proteins, released into the extracellular environment, play key roles in microbial growth, metabolism, environmental adaptation, and host interactions. Among the 75 Ascomycota strains, the number of secreted protein-coding genes ranged from 195 to 1380, representing 2.1% to 9.8% of all coding genes. Compared to PF and SF, EF showed significant expansion of secreted protein-coding gene families (Figure 9A). This expansion was most pronounced in EFR (*p* < 0.05, pairwise PERMANOVA). Variation in the number of secreted protein-coding genes was primarily influenced by phylogeny and lifestyle, with phylogeny being the dominant factor (contribution rate: 18.0%; *p* < 0.05, pairwise PERMANOVA; Figure 9E). Small secreted proteins (SSPs) constituted 24.0–59.4% of all secreted proteins, forming a major component of the secretome. Although the number of SSP-coding genes did not differ significantly across the three lifestyles overall (Figure 9C), it was significantly higher in EFR than in PF, SF, and EFNR, indicating a specific expansion of SSP gene families in root endophytes (*p* < 0.05, pairwise PERMANOVA). Variation in the number of SSP-coding genes was significantly influenced by phylogeny (contribution rate: 14.0%; *p* < 0.05, pairwise PERMANOVA; Figure 9G). We next compared the abundance of secreted proteases and CAZymes across the three lifestyles. Compared to PF and SF, EF exhibited significant expansion of both secreted protease and CAZyme gene families (*p* < 0.05, pairwise PERMANOVA; Figure 9B,D). EFR showed the most pronounced expansion and the highest average abundance. The abundances of secreted proteases and CAZymes were both significantly influenced by phylogeny and lifestyle (*p* < 0.05, pairwise PERMANOVA). Secreted protease abundance was primarily influenced by phylogeny (contribution rate: 19.0%; Figure 9F), whereas secreted CAZyme abundance was mainly affected by lifestyle (contribution rate: 18.3%; Figure 9H).

Despite the overall expansion of the secretome in EF, we observed considerable variation within this group. In Figure 9A, the majority of EF strains clustered above the PF and SF averages, consistent with the significant expansion described above. However, several EF strains fell below the PF/SF average, falling within the range typical of pathogens or saprophytes. These outlier strains include members of the genera *Xylaria*, *Daldinia*, and *Aspergillus* (e.g., *Xylaria multiplex* DSM 110363, *Daldinia* sp. EC12, and *Aspergillus aculeatinus* CBS 121060). Notably, *Xylaria* and *Daldinia* are wood-associated fungi commonly isolated as endophytes from woody hosts, while the Aspergillus outlier represents an EFNR. The reduced secretome size in these outliers may reflect alternative ecological strategies: wood-associated endophytes might rely more on secondary-metabolite production or enzymatic cocktails specialized for lignocellulose degradation rather than a broad spectrum of secreted proteins. These observations suggest that the endophytic lifestyle encompasses distinct subclasses with divergent genomic configurations, warranting further investigation into the functional implications of secretome variation within this group.

**Figure 10:**
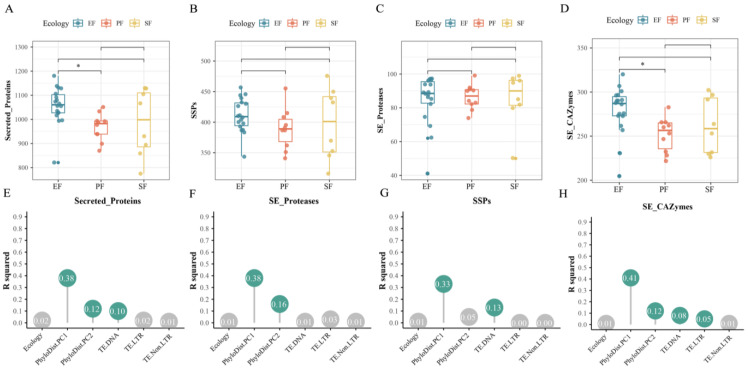
Expansion of the secretome in *Fusarium* endophytes and its drivers (*, *p* < 0.05, pairwise PERMANOVA). Abundance of secreted protein (**A**), secreted protease (**B**), SSP (**C**), and secreted CAZyme (**D**) gene families across lifestyles; (**E**–**H**) contribution of genomic features to the variation in each corresponding gene family above.

For the 36 *Fusarium* strains, we predicted a total of 36,596 secreted proteins, which included 14,473 SSPs. Within the secretome, secreted protein-coding genes accounted for 6.0–7.4% of all genes, and SSPs comprised the majority (61.8–82.5%) of secreted proteins. EF had the highest average number of secreted proteins, which was significantly greater than that of PF (*p* < 0.05, pairwise PERMANOVA; Figure 10A). Variation in the number of secreted protein-coding genes was primarily influenced by phylogeny and TE content, with phylogeny being the dominant factor (contribution rate: 50.2%; *p* < 0.05, pairwise PERMANOVA; Figure 10E). Although the number of SSP-coding genes did not differ significantly across lifestyles overall (Figure 10C), EF had a higher average abundance. Notably, EFR showed the highest average abundance, which was significantly greater than that of PF and EFNR (*p* < 0.05, pairwise PERMANOVA). Variation in the number of SSP-coding genes was significantly influenced by phylogeny and TE content, with phylogeny as the dominant factor (contribution rate: 33.2%; *p* < 0.05, pairwise PERMANOVA; Figure 10G). We also compared the abundance of proteases and CAZymes within the secretome across lifestyles. The number of secreted protease gene families did not differ significantly across lifestyles (Figure 10B). In contrast, EF had significantly more CAZyme gene families than PF (*p* < 0.05, pairwise PERMANOVA; Figure 10D). EFR, in particular, exhibited significant expansion of these gene families, showing higher abundance than PF, SF, and EFNR. Variation in the number of secreted protease gene families was significantly influenced by phylogeny (contribution rate: 53.6%; *p* < 0.05, pairwise PERMANOVA; Figure 10F). Variation in the number of CAZyme gene families was significantly influenced by both phylogeny and TE content, with phylogeny being the dominant factor (contribution rate: 53.3%; *p* < 0.05, pairwise PERMANOVA; Figure 10H).

The differential contributions of lifestyle and phylogeny between datasets and across gene families reveal important biological patterns. In the Ascomycota dataset, lifestyle explained a significant proportion of the variance in secreted-CAZyme content (18.3%, Figure 9H), but its effect on secreted-protease abundance was not significant in the multivariate context (Figure 9F). This suggests that different functional components of the secretome are under distinct evolutionary constraints: secreted CAZymes, which are directly involved in plant cell wall degradation and host interaction, may be more responsive to ecological niche shifts, whereas secreted proteases might be more constrained by phylogenetic heritage. In contrast, for the Fusarium dataset, phylogeny dominated the variation in both secreted proteases and CAZymes (contributing >50%, Figure 10F,H), reflecting the close relatedness of strains within this genus. Here, lifestyle effects were only detectable for secreted CAZymes (Figure 10H), possibly because recent ecological diversification within Fusarium has primarily shaped the CAZyme repertoire rather than proteolytic functions.

The relative prominence of PhyloDist.PC1 versus PhyloDist.PC2 across analyses further illustrates the complexity of phylogenetic effects. In the Ascomycota, PhyloDist.PC1 (representing deep evolutionary splits) often explained a larger share of variance in traits that are conserved across major lineages, while PhyloDist.PC2 (capturing more recent divergences) tended to correlate with lifestyle-associated gene families (e.g., Figure 9H). In the Fusarium dataset, where all strains share a recent common ancestor, the phylogenetic signal is compressed, and PhyloDist.PC1 alone accounted for most of the residual phylogenetic variation, making it the dominant covariate in all models (Figure 10E–H). These patterns underscore the importance of accounting for phylogenetic scale when interpreting lifestyle–trait associations.

#### 3.3.2. Comparative Analysis of CAZyme Gene Families

CAZymes play crucial roles in fungus–plant interactions, facilitating fungal nutrient acquisition and infection, while also influencing plant defense and ecosystem nutrient cycling. To systematically characterize the CAZyme repertoires associated with different lifestyles, we analyzed the abundance of CAZyme gene families in both the Ascomycota and Fusarium datasets, with a focus on total CAZymes, PCWDEs, and MCWDEs.

**Figure 11:**
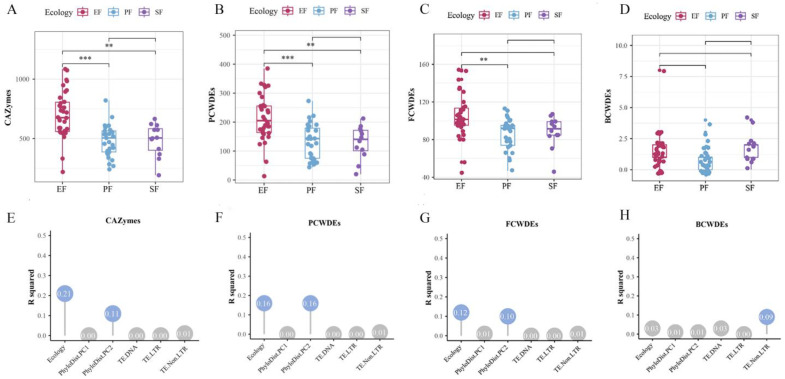
Expansion of the CAZyme in Ascomycota endophytes and its drivers (**, *p* < 0.01; ***, *p* < 0.001; pairwise PERMANOVA). Abundance of CAZyme (**A**), PCWDE (**B**), FCWDE (**C**), and BCWDE (**D**) gene families across lifestyles; (**E**–**H**) contribution of genomic features to the variation in each corresponding gene family above.

In the 75 Ascomycota strains, the number of CAZyme-coding genes ranged from 191 to 1085. Compared to PF and SF, EF showed significant expansion of CAZyme gene families (Figure 11A,E). EFR harbored significantly more CAZyme gene families than PF, SF, and EFNR (*p* < 0.05, pairwise PERMANOVA). This expansion was primarily driven by auxiliary activity (AA) gene families. Among the major CAZyme classes, AA, carbohydrate esterase (CE), glycoside hydrolase (GH), and polysaccharide lyase (PL) gene families were significantly expanded in EF (*p* < 0.05), whereas glycosyltransferase (GT) families showed no significant differences across lifestyles. PCWDEs constituted 6.0–36.4% of all CAZymes. EF exhibited significant expansion of PCWDE gene families (*p* < 0.05; Figure 11B), with EFR showing the most pronounced expansion. Variation in PCWDE numbers was significantly influenced by phylogeny and lifestyle (*p* < 0.05), with phylogeny being the primary factor (contribution rate: 16.3%; Figure 11F). EF also showed significant expansion of PCWDE families targeting cellulose, hemicellulose, lignin, and pectin. The AA9 family was the most abundant among cellulose-degrading CAZymes, and EF harbored significantly more AA9 genes than PF. For hemicellulose degradation, EF displayed expansion of CE3 and CE4 families; differences between EFR and EFNR were largely attributable to GH2 families. EF also showed expansion of pectin-degrading families (GH78, CBM67) and the lignin-degrading AA2 family. MCWDEs, comprising fungal FCWDEs and BCWDEs, were also examined. EF, and particularly EFR, had the highest average number of FCWDE-coding genes (Figure 11C), with significant expansion of FCWDEs targeting chitin and glucan degradation. Variation in FCWDE numbers was significantly influenced by lifestyle (*p* < 0.05; Figure 11G). In contrast, BCWDE-coding genes did not differ significantly across lifestyles, although SF had the highest average (Figure 11D,H).

**Figure 12:**
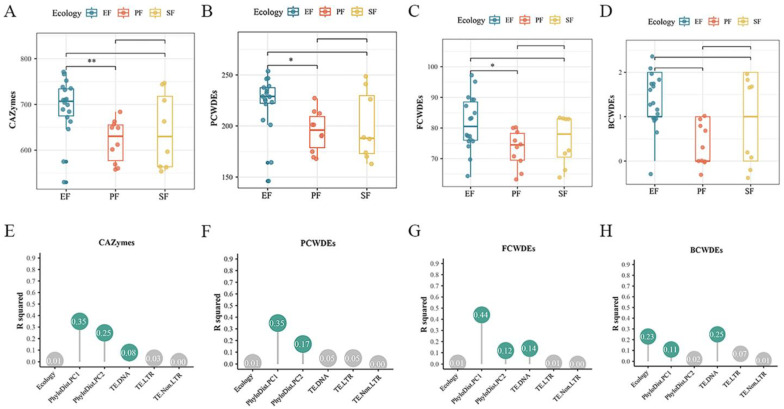
Expansion of the CAZyme in *Fusarium* endophytes and its drivers (*, *p* < 0.05; **, *p* < 0.01; pairwise PERMANOVA). Abundance of CAZyme (**A**), PCWDE (**B**), FCWDE (**C**), and BCWDE (**D**) gene families across lifestyles; (**E**–**H**) contribution of genomic features to the variation in each corresponding gene family above.

In the 36 *Fusarium* strains, the number of CAZyme families ranged from 530 to 771. EF had the highest average number of CAZyme gene families (Figure 12A,E). EFR possessed significantly more CAZyme families than PF, SF, and EFNR, indicating a marked expansion. Within the proteome, EF had the highest average numbers of AA, carbohydrate-binding module (CBM), GH, and GT families, all significantly more abundant than in PF. Variation in CAZyme numbers was significantly influenced by phylogeny and DNA transposon content (*p* < 0.05). PCWDEs accounted for 27.5–33.8% of total CAZymes. EF had significantly more PCWDE families than PF (*p* < 0.05). EFR showed the highest average number of PCWDE families (Figure 12B), and variation was primarily influenced by phylogeny (contribution rate: 52.2%; Figure 12F). No significant differences were detected across lifestyles in PCWDE families dedicated to hemicellulose or lignin degradation. Cellulose-degrading CAZymes were dominated by AA and GH families, with AA9 being the most abundant. EF exhibited significant expansion of GH1 and GH7 families (*p* < 0.05). The pectin-degrading GH28 and GH78 families showed significant expansion, and their variation was significantly influenced by DNA transposon content (*p* < 0.05). For MCWDEs, EF had the highest average number of FCWDE families (Figure 12C), with EFR showing the most significant expansion, particularly in families targeting chitin, mannan, and glucan degradation. EFR also had the highest average number of BCWDE families (Figure 12D). DNA transposon content significantly influenced the distribution of both BCWDE and FCWDE families (Figure 12G,H).

#### 3.3.3. Expansion of Virulence Factors

We systematically analyzed the distribution of virulence factors across the three fungal ecotypes using the DFVF and PHI databases. Data from both databases were manually curated to ensure reliability. To improve annotation accuracy, virulence factor predictions from DFVF were validated by BLAST v2.2.31 against the PHI database.

**Figure 13:**
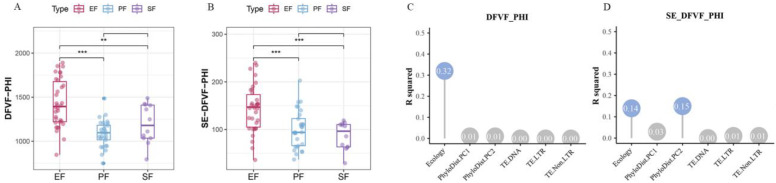
Expansion of the virulence factors in Ascomycota endophytes and its drivers (**, *p* < 0.01; ***, *p* < 0.001; pairwise PERMANOVA). Abundance of virulence factors (**A**) and secreted virulence factors (**B**) coding gene families across lifestyles; (**C**,**D**) contribution of genomic features to the variation in each corresponding gene family above.

Among the 75 Ascomycota strains, EF harbored the highest number of virulence factor-coding genes. This was especially true for EFR, which showed significant differences compared to both PF and SF (*p* < 0.05, pairwise PERMANOVA; Figure 13A,C). As expected, PF possessed more secreted-virulence factor gene families on average than SF, which had the lowest numbers (Figure 13B,D). Several CAZyme families function as virulence factors in plant cell wall degradation. We functionally annotated virulence factor-coding genes to identify those encoding CAZymes involved in plant cell wall degradation. Among the Ascomycota strains, CAZymes constituted a relatively small proportion (8–19%) of all virulence factors in the proteome. This proportion increased substantially in the secretome (25–75%). This indicates that most CAZyme-derived virulence factors are secreted. EF exhibited significant expansion of genes encoding CAZyme-based virulence factors (*p* < 0.05, pairwise PERMANOVA). Differences in the abundance of these CAZyme-based virulence genes among EF, PF, and SF were largely driven by variation in the AA, CE, GH, and PL families. Among these, the AA, CE, and GH families were the most abundant. SF had the lowest average numbers of AA, CE, and PL gene families. Specific subfamilies, such as AA1_3, CE5, and CE8, were significantly less abundant in SF than in EF and PF. EF showed expansion of the AA7, CE5, and CE8 families, while EFR were particularly enriched for the AA9 family. The AA9 family degrades cellulose, facilitates plant cell wall deconstruction, and promotes fungal infection [44]. Most PL family virulence factors are secreted. Among the major subfamilies, EF showed expansion of PL3_2 but not PL1_7. The PL1_7 family plays a key role in endophyte infection and host colonization [45]. The AA, CE, and PL families are thus important for fungal host infection.

**Figure 14:**
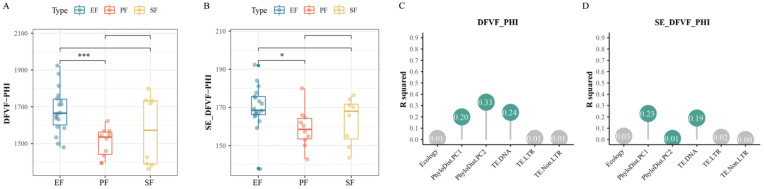
Quantitative difference in virulence factor-coding genes of 36 *Fusarium* fungi and corresponding contribution degree of genomic characteristics. Expansion of the virulence factors in *Fusarium* endophytes and its drivers (*, *p* < 0.05; ***, *p* < 0.001; pairwise PERMANOVA). Abundance of virulence factors (**A**) and secreted virulence factors (**B**) coding gene families across lifestyles; (**C**,**D**) contribution of genomic features to the variation in each corresponding gene family above.

In the *Fusarium* proteome, EF had a significantly higher average number of virulence factor-coding genes than PF (*p* < 0.05, pairwise PERMANOVA; Figure 14A,C). In the secretome, however, the number of virulence factor gene families did not differ significantly across lifestyles, despite EF (and particularly EFR) having the highest average counts (Figure 14B,D). In the 36 *Fusarium* strains, over 50% of genes encoding CAZyme-based virulence factors were predicted to be secreted, consistent with the Ascomycota results. Within the secretome, EFR showed significant expansion of genes encoding CAZyme-based virulence factors (*p* < 0.05, pairwise PERMANOVA). EF showed expansion of the AA7, AA1_3, GH28, and GH3 families. EFR specifically showed expansion of AA1_3, GH3, and GT1. The GH3 family, encoding β-glucosidases, plays a key role in fungal growth and host infection by hydrolyzing β-1,4-glycosidic bonds in plant cell wall polysaccharides such as cellulose and hemicellulose [46]. These CAZyme families thus function as virulence factors and are crucial for fungus–plant interactions.

## 4. Discussion

Our comparative genomic analyses challenge the prevailing notion that the evolution of a symbiotic lifestyle in fungi is predominantly characterized by genomic reduction and metabolic simplification, as exemplified by obligate mutualists like ectomycorrhizal fungi [16,17,18,19]. Instead, we unveil a distinct evolutionary trajectory in Ascomycota endophytes: one marked by TE-driven genome expansion and the retention and expansion of a broad genetic toolkit that encompasses capabilities for both saprotrophic decomposition and host interaction.

Ascomycota fungi exhibit substantial genetic and lifestyle diversity. EF are phylogenetically diverse, spanning multiple orders [47,48], and significantly influence plant health, growth, and microbial community structure. Although the genomic mechanisms underlying endophytic adaptation are not fully understood, previous studies highlight their functional roles. For example, Khalmuratova et al. [49] showed that endophytes from saline environments (e.g., *Suaeda australis*) produce phytohormones that promote plant growth. Similarly, the endophyte *Talaromyces omanensis* enhances drought tolerance in its host by increasing chlorophyll content and potassium uptake [49]. Furthermore, endophytes from medicinal plants are prolific producers of bioactive secondary metabolites [50,51].

Our analysis reveals that EF possess higher TE content than other lifestyles, with a notable enrichment of DNA transposons in EFR. We found a significant positive correlation between DNA transposon content and genome size, suggesting that TE accumulation drives genome expansion in EF. Moreover, the number of secondary-metabolite biosynthetic gene clusters also correlated with TE content. EF harbored the highest number of these clusters, a trait potentially linked to TE accumulation, as reported in other systems [52]. This phenomenon is well-documented; for instance, genome expansion via TE accumulation has been reported in ectomycorrhizal taxa across diverse lineages including *Amanita*, Hymenochaetales, Russulales, and *Fusarium* [16,53,54,55].

In contrast to the extensive loss of PCWDE genes observed in ectomycorrhizal fungi [56,57]. Schlegel et al. [58] reported expansion of CAZyme gene families in the root endophyte *Phialocephala subalpina*. Similarly, Martino et al. [59] found that ericoid mycorrhizal fungi retain substantial CAZyme complements. Almario et al. [60] also linked an endophytic lifestyle with CAZyme expansion, specifically in FCWDEs. Consistent with these reports, we found that EF (especially EFR) exhibit significant expansion, not loss, of CAZyme gene families. This includes significant expansion of PCWDE families targeting cellulose, hemicellulose, and pectin. In *Fusarium*, however, expansion was more specific (e.g., pectin-degrading PCWDEs), indicating dynamic, lineage-specific changes in the CAZyme repertoire during adaptation, while core functions like FCWDEs were retained. Crucially, this expansion of the saprotrophic arsenal, particularly in EFR, showed a significant positive correlation with DNA transposon content. This suggests that TE-mediated genomic plasticity may have facilitated the duplication and neofunctionalization of key CAZyme genes, providing the raw material for enhanced decomposing ability while inhabiting a living host.

Beyond PCWDEs, EF also expanded other gene families involved in organic matter decomposition, including proteases and FCWDEs. Notably, in *Fusarium*, the genomic potential for saprotrophy in EF correlates with TE content. This supports a dual endophytic–saprotrophic strategy in EF, necessitating a broad arsenal of degradative enzymes, whose expansion may be facilitated by TE accumulation.

The functions of SSPs are essential for infecting the host and establishing endophytic symbiosis [61]. We observed expansion of SSP gene families in EFR, notably CSEPs, which are likely key for successful host colonization. The expansion of SSP families correlated significantly with TE content, particularly DNA transposons. Furthermore, EF possess more virulence factor-encoding genes than PF or SF, with EFR harboring the highest numbers. This retention of a substantial virulence repertoire suggests a latent pathogenic potential in EF. Supporting this idea, Hiruma et al. [62] showed that the root endophyte *Colletotrichum tofieldiae* can dynamically shift along the parasite–mutualist spectrum by regulating secondary-metabolite clusters. Furthermore, TEs are frequently found in proximity to genes encoding SSPs and can drive the evolution of virulence-related factors, which in turn aids host infection [63,64].

Our findings also offer a genomic perspective on the well-documented phenotypic plasticity of endophytic fungi. The observation that EF genomes are significantly expanded and enriched for gene families associated with both saprotrophic decomposition and host interaction or virulence is consistent with the presence of a versatile genomic toolkit that may facilitate lifestyle flexibility. This “dual-trophic potential,” defined as the retention of genetic machinery for both mutualistic and antagonistic interactions, may provide a partial explanation for how a single fungal genotype could shift along the symbiotic–parasitic continuum in response to host physiology or environmental stress. Rather than requiring de novo mutations for each lifestyle transition, the necessary genetic repertoire appears to be already present; its differential expression or regulation, potentially influenced by epigenetic changes associated with TEs, might play a role in such ecological transitions. Therefore, the TE-driven genomic plasticity we observe may represent more than just genome size expansion; it could contribute to the raw material for the evolution and maintenance of the phenotypic plasticity that characterizes the endophytic lifestyle.

Integrating these findings, we propose a novel evolutionary model for facultative endophytism in Ascomycota: Transposable Element-Driven Genomic and Ecological Plasticity. In this model, bursts of TE activity, particularly of DNA transposons, drive genome expansion and instability. This plasticity, in turn, fosters the retention, duplication, and diversification of gene families underlying both saprotrophic nutrition and host manipulation. The resultant “genomic toolbox” equips these fungi with dual-trophic capabilities, allowing them to opportunistically switch between endophytic, saprophytic, and latent pathogenic modes depending on host status and environmental conditions. This model reframes endophytes not merely as symbiotic specialists but as versatile ecological opportunists, whose adaptive potential is deeply rooted in a TE-malleable genome.

## Data Availability

The data presented in this study are available in the National Center for Biotechnology Information GenBank database (https://www.ncbi.nlm.nih.gov/genbank/, accessed on 10 March 2025).

## Abbreviations

The following abbreviations are used in this manuscript:

AA	Auxiliary Activity
BCWDE	Bacterial Cell Wall-Degrading Enzyme
BUSCO	Benchmarking Universal Single-Copy Orthologs
CAZymes	Carbohydrate-Active Enzymes
CBM	Carbohydrate-Binding Module
CE	Carbohydrate Esterase
CSEP	Candidate Secreted Effector Protein
DFVF	Database of Fungal Virulence Factors
EF	Endophytic Fungi
EFNR	Non-Root-Endophytic Fungi
EFR	Root-Endophytic Fungi
FCWDEs	Fungal Cell-Wall-Degrading Enzymes
GH	Glycoside Hydrolase
GT	Glycosyl Transferase
HMM	Hidden Markov Model
LiP	Lignin Peroxidase
LTR	Long Terminal Repeat
MCWDEs	Microbial Cell Wall-Degrading Enzymes
MnP	Manganese Peroxidase
NCBI	National Center for Biotechnology Information
non-LTR	Non-Long Terminal Repeat
PCWDE	Plant Cell Wall-Degrading Enzyme
PERMANOVA	Permutational Multivariate Analysis of Variance
PF	Pathogenic Fungi
PHI	Pathogen–Host Interaction database
PL	Polysaccharide Lyase
SF	Saprotrophic Fungi
SSP	Small Secreted Protein
TE	Transposable Element
TRF	Tandem Repeats Finder

## Appendix A

**Table A1:** Taxonomic information of 75 Ascomycota strains.

GeneBank Assembly	Ecology	Strains
GCA_020744275.1	EF (EFR)	*Macrophomina phaseolina* MPI-SDFR-AT-0080
GCA_000340195.1	PF	*Dothistroma septosporum* NZE10
GCF_900074925.1	PF	*Ramularia collo-cygni* URUG2
GCF_000320565.1	PF	*Sphaerulina musiva* SO2202
GCA_010093825.1	PF	*Teratosphaeria nubilosa* CBS 116005
GCA_020747015.1	EF (EFR)	*Pyrenochaeta* sp. MPI-SDFR-AT-0127
GCF_010094145.1	PF	*Didymella exigua* CBS 183.55
GCA_014281115.1	EF (EFR)	*Laburnicola* sp. R22_1
GCF_000230375.1	PF	*Leptosphaeria maculans* JN3 v23.1.3
GCA_010405375.1	SF	*Lophiostoma macrostomum* CBS 122681
GCA_003073855.1	EF (EFR)	*Periconia macrospinosa* DSE2036
GCA_030544205.1	EF (EFR)	*Paraphoma chrysanthemicola* DS-84
GCA_020744225.1	EF (EFR)	*Paraphoma chrysanthemicola* MPI-GEGE-AT-0034
GCF_000146915.1	PF	*Parastagonospora nodorum* SN15
GCA_009650635.1	EF (EFNR)	*Alternaria alternata* JS-1623
GCA_003574525.	EF (EFNR)	*Alternaria* sp. MG1
GCA_016162275.1	EF (EFR)	*Curvularia geniculata* P1
GCA_004156035.1	PF	*Alternaria tenuissima* FERA 1166
GCF_000523435.1	PF	*Bipolaris zeicola* 26-R-13
GCF_000359705.1	PF	*Exserohilum turcicum* Et28A
GCF_003184765.1	EF (EFNR)	*Aspergillus aculeatinus* CBS 121060
GCA_020826735.1	EF (EFNR)	*Aspergillus montevidensis* ZYD4
GCA_003184635.1	EF (EFNR)	*Aspergillus sclerotiicarbonarius* CBS 121057
GCA_003344595.1	EF (EFNR)	*Penicillium polonicum* hy4
GCA_000600275.1	PF	*Aspergillus ruber* CBS 135680
GCF_014117465.1	SF	*Aspergillus flavus* NRRL3357
GCA_001890685.1	SF	*Aspergillus luchuensis* CBS 106.47
GCA_000269785.2	SF	*Aspergillus oryzae* 3.042
GCF_004022145.1	SF	*Paecilomyces variotii* CBS 101075
GCF_000003125.1	SF	*Talaromyces stipitatus* ATCC 10500
GCF_000149585.1	SF	*Histoplasma mississippiense* NAm1
GCA_001006345.1	PF	*Phaeomoniella chlamydospore* UCRPC4
GCA_000827325.1	EF (EFR)	*Oidiodendron maius* Zn
GCA_003073865.1	EF (EFR)	*Cadophora* sp. DSE1049
GCA_009805495.1	EF (EFNR)	*Pezicula neosporulosa* M44
GCA_900073065.1	EF (EFR)	*Phialocephala subalpina* UAMH 11012
GCA_013461495.1	PF	*Botrytis fragariae* BVB16
GCA_000503235.1	PF	*Sclerotinia borealis* F-4128
GCF_001619985.1	EF (EFNR)	*Xylona heveae* TC161
GCA_003635345.1	SF	*Coniochaeta pulveracea* CAB683
GCA_011745365.1	PF	*Cryphonectria parasitica* EP155
GCA_001630405.1	EF (EFR)	*Diaporthe ampelina* S3MP
GCA_003795295.1	PF	*Cytospora leucostoma* SXYLt
GCA_003795315.1	PF	*Valsa malicola* 03_1
GCA_003795275.1	PF	*Valsa sordida* YSFL
GCA_003568745.1	EF (EFNR)	*Colletotrichum gloeosporioides* ES026
GCA_001563125.1	PF	*Colletotrichum salicis* CBS 607.94
GCF_003724135.2	PF	*Verticillium nonalfalfae* TC161
GCF_012550715.1	PF	*Geosmithia morbida* 1262
GCA_000347355.1	PF	*Claviceps purpurea* 20.1
GCF_000187425.2	PF	*Metarhizium robertsii* ARSEF 23
GCA_001636815.1	PF	*Niveomyces insectorum* RCEF 264
GCA_003025155.1	SF	*Trichoderma longibrachiatum* ATCC 18648
GCA_029931735.1	EF (EFNR)	*Dactylonectria alcacerensis* CT-6
GCA_016952355.1	EF (EFNR)	*Fusarium culmorum* Class2-1B
GCA_014324445.1	EF (EFNR)	*Fusarium oxysporum* Fo47
GCA_016952305.1	EF (EFNR)	*Fusarium pseudograminearum* Class2-1C
GCA_954870535.1	EF (EFNR)	*Fusarium* sp. VM40
GCA_003934905.1	EF (EFNR)	*Neonectria* sp. DH2
GCA_020744385.1	EF (EFR)	*Fusarium flagelliforme* MPI-CAGE-AA-0113
GCA_020744495.1	EF (EFR)	*Fusarium solani* FSSC 5 MPI-SDFR-AT-0091
GCA_001292635.1	PF	*Fusarium langsethiae* Fl201059
GCA_008271525.1	EF (EFNR)	*Sarocladium brachiariae* HND5
GCF_000002495.2	PF	*Pyricularia oryzae* 70-15
GCA_000968615.1	PF	*Thielaviopsis punctulata* CR-DP1
GCF_000143105.1	PF	*Grosmannia clavigera* kw1407
GCF_000226545.1	SF	*Podospora anserina S mat+*
GCF_000182925.2	SF	*Neurospora crassa OR74A*
GCA_002120325.1	EF (EFNR)	*Daldinia* sp. EC12
GCA_002120315.1	EF (EFNR)	*Hypoxylon* sp. CI-4A
GCA_002120335.1	EF (EFNR)	*Hypoxylon* sp. EC38
GCA_001566295.1	EF (EFR)	*Microdochium bolleyi* J235TASD1
GCF_000516985.1	EF (EFNR)	*Pestalotiopsis fici* W106-1
GCA_001445595.3	PF	*Rosellinia necatrix* W97
GCA_011057905.1	SF	*Xylaria multiplex* DSM 110363

**Table A2:** Taxonomic information of 36 *Fusarium* fungal strains.

GeneBank Assembly	Ecology	Strains
GCA_014325065.1	EF (EFR)	*F. oxysporum* Fo16
GCA_016166015.1	SF	*F. oxysporum* GL1552
GCA_020744455.1	EF (EFR)	*F. oxysporum* MPI-SDFR-AT-0094
GCA_014324665.1	EF (EFR)	*F. oxysporum* Fo45
GCA_040285575.1	EF (EFNR)	*F. oxysporum* 1LF1-1
GCA_040285555.1	EF (EFR)	*F. oxysporum* 8RF1-3
GCA_014325295.1	EF (EFR)	*F. oxysporum* Fo2
GCA_014325035.1	SF	*F. oxysporum* Fo12
GCA_020744355.1	EF (EFR)	*F. oxysporum* MPI-CAGE-CH-0212
GCA_020744335.1	EF (EFR)	*F*. sp. MPI-SDFR-AT-0072
GCA_013755755.1	SF	*F*. sp. NRRL 25184
GCA_022627115.1	EF (EFNR)	*F. annulatum* FFSC RH5
GCA_900067095.1	EF (EFR)	*F. proliferatum* ET1
GCA_900096505.1	PF	*F. fujikuroi* B14
GCA_022627135.1	EF (EFNR)	*F. proliferatum* FFSC RH7
GCA_022627125.1	EF (EFNR)	*F. chuoi* FFSC RH1
GCA_019915245.1	PF	*F. musae* F31
GCA_037214365.1	PF	*F. verticillioides* ZH12-2
GCA_013781345.1	EF (EFNR)	*F. coicis* NRRL 66233
GCA_040114195.1	PF	*F. circinatum* Fc25332
GCA_002980515.1	SF	*F. burgessii* NRRL 66654
GCA_033439405.1	EF (EFNR)	*F*. sp. NFCCI 5145
GCA_001717915.1	SF	*F. graminearum* DAOM180378
GCA_025427445.1	PF	*F. graminearum* FG187
GCA_025427465.1	PF	*F. asiaticum* FA031
GCA_012600195.1	PF	*F. cerealis* Fcer1134NY13
GCA_001567575.1	PF	*F. sambucinum* F-4
GCA_019425555.1	SF	*F. venenatum* A3/5
GCA_003012285.1	SF	*F. longipes* NRRL 20695
GCA_003313175.1	PF	*F. equiseti* D25-1
GCA_025948275.1	PF	*F. avenaceum* WV21P1A
GCA_954870535.1	EF (EFNR)	*F*. sp. VM40
GCA_021655875.1	SF	*F. guadeloupense* NRRL 36125
GCA_027946385.1	EF (EFNR)	*F. piperis* IMI 366586
GCA_013168735.1	EF (EFNR)	*F. solani* IISc-1
GCA_033030565.1	EF (EFR)	*F. nematophilum* NQ8GII4
